# Sample-Specific Prediction Error Measures in Spectroscopy

**DOI:** 10.1177/0003702820913562

**Published:** 2020-05-26

**Authors:** Carl Emil Eskildsen, Tormod Næs

**Affiliations:** 1 Nofima AS, Norwegian Institute for Food, Fisheries and Aquaculture Research, Ås, Norway; 2 Institute for Biodiversity and Ecosystem Dynamics, University of Amsterdam, Amsterdam, The Netherlands

**Keywords:** Multivariate calibration, sample-specific uncertainty, spectroscopy, principal component regression, PCR

## Abstract

In applied spectroscopy, the purpose of multivariate calibration is almost exclusively to relate analyte concentrations and spectroscopic measurements. The multivariate calibration model provides estimates of analyte concentrations based on the spectroscopic measurements. Predictive performance is often evaluated based on a mean squared error. While this average measure can be used in model selection, it is not satisfactory for evaluating the uncertainty of individual predictions. For a calibration, the uncertainties are sample specific. This is especially true for multivariate calibration, where interfering compounds may be present. Consider in-line spectroscopic measurements during a chemical reaction, production, etc. Here, reference values are not necessarily available. Hence, one should know the uncertainty of a given prediction in order to use that prediction for telling the state of the chemical reaction, adjusting the process, etc. In this paper, we discuss the influence of variance and bias on sample-specific prediction errors in multivariate calibration. We compare theoretical formulae with results obtained on experimental data. The results point towards the fact that bias contribution cannot necessarily be neglected when assessing sample-specific prediction ability in practice.

## Introduction

Prediction uncertainty estimation is important for instance when using spectroscopic measurements for telling the state of a chemical reaction or doing process control.^1^ In such cases, a calibration model is fitted using a set of spectroscopic measurements with corresponding reference values. When applying the calibration model, for example during production, reference values are (normally) not available. Hence, one must solely rely on predicted values when controlling the process. In such situation, good estimates of sample-specific prediction errors are necessary to judge the validity of the prediction. In this paper, we compare sample-specific prediction errors obtained from experimental data with the sample-specific errors derived from theoretical formulae.

The most frequently used methods for investigating the reliability of calibration models are cross-validation based on the calibration data itself or prediction testing based on independent dataset(s).^2^ Both these methods provide information on average prediction abilities over the samples tested, expressed as, e.g., a mean squared error (MSE). But the methods give no information about how prediction ability changes across samples. It is known, both theoretically and in practice, that the best predictions are obtained in the center of the calibration data and also that the prediction ability may decrease substantially as one moves away from this center.^3^ Therefore, estimating the prediction ability of a calibration model by means of cross-validation or prediction testing is not fully satisfactory and there is a need for methods, which can give information on sample-specific errors.^4^

For least squares linear regression, the situation is quite simple as the predictor in a linear model is unbiased and the variance of a prediction is easy to calculate using the formula^5^

(1) E(y^-y) 2=σ2+σ2N+σ2xT(XTX) -1x

in which $\sigma^{2}$ is the random error of the linear regression model, *N* is the number of calibration samples, **X** is the centered calibration data (measurements), and **x** represents the measurement (centered according to the calibration data), for which one wishes to obtain the prediction, y^. The true reference value for the sample of interest is given by *y*. As can be seen from Eq. 1, prediction error changes with the values of **x**.

However, due to collinearity in spectral data, data compression methods such as partial least squares (PLS) regression or principal component regression (PCR) must be used, and Eq. 1 cannot be applied directly when dealing with spectral measurements. A good and pragmatic workaround is to use component scores, obtained from, e.g., PCR, rather than the spectral measurements in Eq. 1. However, predictions obtained from PCR (and PLS) are biased due to omitting components in the model.^6^ This bias is not accounted for in Eq. 1 and therefore, Eq. 1 is not satisfactory for the case of PCR (and PLS) as will be shown in this paper.

Since Eq. 1 is obtained by applying standard formulae for variance of linear combinations of a fixed vector, **x**, and a random regression vector, **b**, an alternative possibility when other calibration methods are used is to replace $(X^{\text{T}}X){\;}^{-1}\sigma^{2}$ (the covariance matrix of **b**) with a bootstrap alternative and substitute this alternative in the place of $(X^{T}X){\;}^{-1}\sigma^{2}$ in Eq. 1. This approach can be used for any calibration method.^4^

Faber and Kowalski^7^ based the sample-specific variance of a prediction on the errors in **x**, *y*, and **b** as well as $\sigma^{2}$. This is useful when comparing error contributions. The expression by Faber and Kowalski is also the basis for studies of Andersen and Bro^8^ and Skou et al.^1^

Other approaches can be found in Fernandez-Ahumada et al.^9^ and Zang and Fearn.^10^ Fernandez-Ahumada et al.^9^ handle uncertainties in input data in an error-in-variable context giving rise to an alternative expression for prediction error. Zang and Fearn^10^ use an approximation procedure for estimating the prediction variance for PLS regression.

Nevertheless, the above-mentioned studies put little emphasis on the bias contribution. In this paper, we will use the prediction error formulae for PCR (see Mandel^11^ or Næs and Mevik^5^) as a basis for discussing sample dependent prediction errors more generally. This leads to an investigation of the relative size of the variance and bias contribution for different number of components included in the model. It will be shown that the bias may play an important role in addition to the variance as represented in Eq. 1. In particular, it is important to take the bias into consideration if the number of relevant components is not selected in a satisfactory way. As a part of the discussion, we will distinguish between three different types of bias, namely the omitted-variables bias, the least squares effect bias and the bias occurring when the calibration samples are not representative for the predicted sample. The three types of bias are explained in further details in the Materials and Methods section below.

The error and bias formulae for PCR will be compared with the true squared error (y^-y) 2 and true bias (y^-y) in a prediction testing situation. To the authors’ knowledge, this has not been done before. This investigation has two scopes: First of all, it will be a check of the realism of the theoretical formula in real prediction situations. Secondly, it will be an investigation of the true variability of a prediction error around the estimate given by the formulae.

The structure of the observed errors as a function of the formulae will be studied using Loess, which is a nonparametric regression method useful for indicating tendencies.^12^

In addition, there will be a discussion of the different phenomena involved in prediction error estimation, these results also point towards the fact that the bias contribution cannot necessarily be neglected when assessing prediction ability and variability in practice for each new sample measured.

## Materials and Methods

### Model and Estimation

The data, spectra, and chemical concentrations, for calibration are given by X(N×K) and y(N×1), respectively. The focus here is on building a prediction equation for **y** based on **X**, using a linear model given by

(2) $$y=1b_{0}+\mathrm{Xb}+e$$

where 1(N×1) is a column vector of ones, $b_{0}(1\times 1)$ is the offset, and e(N×1) is the error. Note that $\mathrm{var}(e)=\sigma^{2}$ is the same as in Eq. 1 and denotes the random error of the model. Since the variables (columns) of **X** are highly collinear for applications in spectroscopy, one needs a data compression method such as PCR or PLS. For calibration, one usually assumes that both **X** and **y** are centered column-wise. When using the model for prediction of a new sample, x(1×K), one centers **x** according to **X** and usually adds the original mean of **y** to the prediction.

The procedure used for PCR is based on the singular value decomposition of **X**

(3) $$X={\mathrm{USP}}^{\text{T}}$$

Here, columns of U(N×M) are the left singular vectors of **X**, S(M×M) is a diagonal matrix containing singular values and columns of P(K×M) are the right singular vectors of **X**. Here, *M* denotes the number of non-zero singular values. Both **U** and **P** are orthonormal. Using all the singular vectors **U**, the model in Eq. 2 can be reformulated as

(4) $$y=1g_{0}+\mathrm{Ug}+e$$

where $g_{0}(1\times 1)$ is the offset and g(M×1) are the regression coefficients. For PCR, one uses a limited number of components, *A*, determined by for instance cross-validation, i.e., one uses the reduced model

(5) $$y=1g_{0}+U_{A}g_{A}+f$$

Here, $U_{A}(N\times A)$ is defined as the first *A* columns of **U**, corresponding to the *A* largest eigenvalues of $X^{\text{T}}X$, $g_{A}(A\times 1)$ are the first *A* regression coefficients, and f(N×1) is the error. For a new sample to be predicted, one projects the new sample, **x** (after centering) onto ${\mathrm{PS}}^{-1}$ (Eq. 3) to calculate the corresponding u(1×M) and then uses the first *A* values of **u** in Eq. 5 with the estimated regression coefficients.

For the closely related PLS regression, the main difference lies in how the **U** is calculated by maximizing the covariance between **y** and linear functions of **X**.

### Validation

The fit of a calibration model (e.g., PCR) is estimated from the residuals (f in Eq. 5) as the mean squared error of calibration (MSEC)

(6) MSEC=∑n=1Nfn2N-K-1=∑n=1N(y^n-yn) 2N-K-1

where *K* is the number of variables in **X**. If *K* is larger than *N*, the fit is typically approximated by replacing *K* with the number of PCR components, *A*. Here, *A* is chosen large enough to expect that components beyond *A* carry no systematic information. This is further discussed in the Results and Discussion section below.

The estimation of prediction ability is most frequently done by cross-validation or prediction testing based on an independent dataset with both **X** and **y** measured. In this study, we use the leave-one-out cross-validation (LOOCV) scheme for model selection.^14^ During LOOCV, the *n*th sample is left out during parameter estimation (Eq. 5). The *n*th sample is then predicted using the estimated parameters. One then typically calculates mean squared error of cross-validation (MSECV) from Eq. 7

(7) MSECV=∑n=1N(y^n-yn) 2N

When doing prediction testing, one estimates the parameters (Eq. 5) on a calibration dataset and then apply the estimated parameters to an independent prediction dataset. One then typically calculates the mean squared error of prediction (MSEP) given by Eq. 8

(8) MSEP=∑n=1NP(y^n-yn) 2NP

where *N_P_* corresponds to the number of samples in the independent prediction dataset. The advantage of these two measures (MSECV and MSEP) is that they are based on direct testing on real data. The cross-validation estimates the average prediction ability of predictors estimated using different subsets of the calibration data. In prediction testing, on the other hand, one tests the properties of a given equation with parameters already estimated. This can be done at any point in time, also after some time of use of the prediction equation. Cross-validation and prediction testing measures are slightly different methods but they seem to be used interchangeably in the literature. The focus in this paper is on prediction testing.

### Prediction Error for PCR

For PCR, the prediction error as a function of **u** for a new sample has a simple formula^5^

(9) E(y^-y) 2=σ2+σ2N+σ2∑a=1Aua2+(-∑m=A+1Mumgm)2

where *u* represents elements of **u** and *g* represents the elements of **g** (Eq. 5). The first term in Eq. 9, $\sigma^{2}$, is from the random error. For estimating the random error in the model given by Eq. 2, one can use the MSEC given by Eq. 6. The subsequent term in Eq. 9 represents the variance contribution (estimation error) from the *A* components used in the PCR model

(10) E(y^-Ey^) 2=σ2N+σ2∑a=1Aua2

and the last term in Eq. 9 is the square of the bias contribution (model error) due to omitting components in the model

(11) E(y^-y)=-∑m=A+1Mumgm

**Figure 1.**
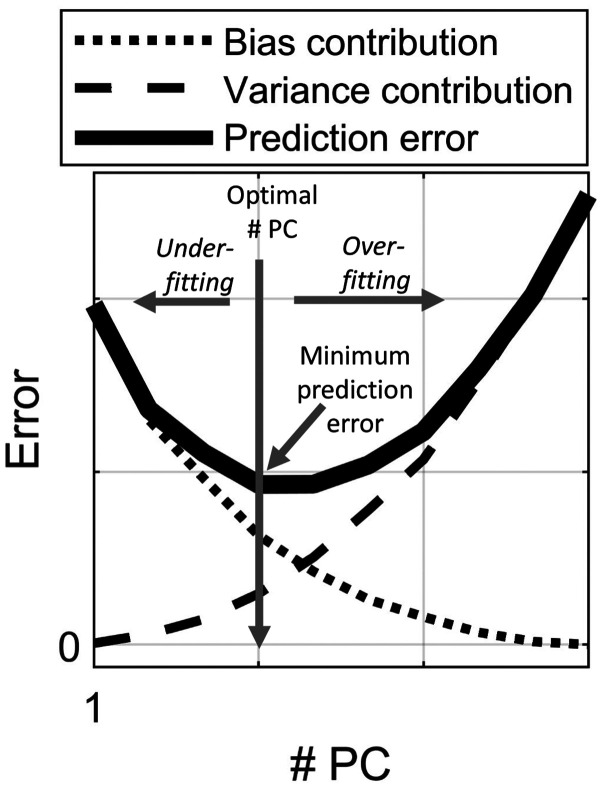
Illustration of the principles underlying predictions error. The variance contribution increases, while the bias contribution decreases when model complexity increases (i.e., when the number of components (# PC) increases).

As seen above, the random error and the variance contribution is similar to the one for least squares regression (Eq. 1), but Eq. 9 also has the bias contribution. The variance contribution increases, whereas the bias contribution decreases with increasing *A* (i.e., more components in the model). In practice, one must balance the two contributions with respect to each other. When the decrease of bias obtained by incorporating a new component is smaller than the extra variance contribution of the same component, it is advantageous to stop incorporating more components in the model. In other words, when the prediction error becomes small enough, it is better to eliminate the components beyond a certain point *A* in order to avoid increased variance. In Fig. 1, this phenomenon is illustrated. Hence, the true prediction error represents a compromise of the variance contribution and the bias contribution. Note that the random error, which is constant, is neglected in Fig. 1.

In the present paper, we will take these formulae (Eqs. 9 to 11) as point of departure and study the relative size of the two contributions as well as how they relate to true errors as measured by (y^-y) 2 and to the true bias measured by (y^-y). For this purpose, estimates of the regression coefficients, g^, are used as the true **g** is unknown. This will provide us with both an indication of the usefulness of the formula and an idea about its precision in practice.

Note that the sample-specific prediction uncertainty can be estimated by plugging in estimates from the calibration. This means that the formulae can be used also in new contexts much later without saving the calibration data.

### Identical Predictions May Have Different Prediction Uncertainty

From Eqs. 5 and 9, it is interesting to note that two samples with the same predicted values y^ can have different prediction errors (y^-y). The reason for this is that different constellations of **u** for two samples (e.g., with variation in quantities of interfering compounds) may return the same predicted values of the analyte of interest (Eq. 5). However, the different constellations of **u** will return different prediction errors (Eq. 9) for the two samples. A demonstration of this will be given, for the example, in this paper.

### Different Types of Bias

It must be noted that bias contribution in Eq. 11 is only one of several possible bias contributions that may occur in practice. In order to clarify this, we will now discuss and distinguish between three different types of bias.

The more intuitive type of bias is present if the calibration samples are not representative for the test samples to be predicted. A typical example can be change of season when estimating for a natural crop or drift of instruments. This type of bias can typically be detected by plotting y^ versus **y**. This bias can take on any shape depending on the situation. In this paper, we will, however, not deal with this type of bias.

The bias described in Eq. 11 is due to omitting principal components in the predictor. As long as components are omitted in a model, this bias will always be there regardless of how many samples are available in the calibration. This bias varies from sample to sample depending on the positioning of *u* along components beyond component *A*. In other words, this bias will not represent a systematic relation between y^ and **y**, and will in practice look like random noise. In the Results and Discussion section, we will, however, present a way to obtain an estimate of its size.

A third and well-known bias is the so-called least squares effect.^13^ This is the bias of y^ as a function of *y* (Eq. 12). Low values/concentrations of a chemical constituent are often overestimated, and the high values are underestimated. This bias is more visible when prediction errors are larger. However, the least square effect bias is always present (like the omitting components bias) even for situations where the parameters, in the linear model, are known exactly. Say the linear predictor $x^{\text{T}}b$ is based on the true regression coefficients in the model, standard distribution theory for the normal distribution gives the following expected value of the predictor y^ conditioned on the value of *y*

(12) y^(y)=bTΣxyΣy-1y

Here, the $\Sigma_{\mathrm{xy}}$ represents the true covariance of **x** and *y* and $\Sigma_{y}^{-1}$ represents the inverse of the variance of *y*. If the sample size is large enough, the covariances and the variance of *y* can be estimated and then used as substitutes for the true values. This will be done in the example below (Results and Discussion section) to visualize the least squares effect bias.

### Data and Preprocessing

The data used for illustration originate from Nielsen et al.^15^ In total, 523 wheat kernels were, individually, measured with near-infrared transmission spectroscopy. Transmission (*T*) was transformed into absorbance by $\text{log}(\frac{1}{T})$ to obtain absorption spectra (**X**). The spectral range, included in this study, was from 860 nm to 1028 nm, with recordings at every second nanometer. Reference variable (**y**) of protein content was determined for each individual wheat kernel using the Kjeldahl analysis. For a detailed sample description, see Nielsen et al.^15^

For calibration, 100 samples were randomly selected, and the remaining 423 samples were used for prediction testing. The major purpose of this study is to compare the prediction error formulae with real prediction errors. Therefore, the majority of samples are in the test set.

Spectra (**X**) were preprocessed by Savitzky–Golay second-order derivative (window size of 21 points and second-order polynomial). Prior to modeling, both **X** and **y** were column-wise mean centered. Data were analyzed in Matlab version R2018a (v.9.4.0.813654, The MathWorks Inc.).

## Results and Discussion

The preprocessed **X** is presented in the supplementary material (Figure S1). Likewise, the results from singular value decomposition of **X** are presented for the first two principal components (Figure S2). Figure S2a shows the loadings, given by **P** in Eq. 3 and Figure S2b shows the scores, given by US in Eq. 3. The decomposition is first calculated on the calibration dataset. Then the prediction dataset is projected onto the model to obtain score values of the 423 prediction samples. By investigating leverages and squared residuals (data not shown), we found that the calibration data are representative for the prediction samples. Hence, a bias is not introduced due to calibration data not being representative for the test data.

**Figure 2.**
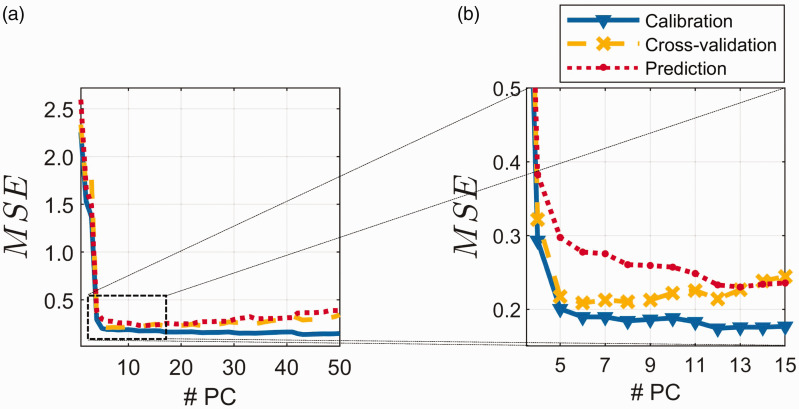
Model fit expressed as mean squared error (MSE) as a function of number of principal components (# PC) in the model. Mean squared error of calibration (blue), mean squared error of cross-validation using the leave-one-out scheme (yellow) and mean squared error of prediction (red). (a) Components 1 to 50. (b) Magnification of the relevant part of (a) (Components 4 to 15).

Figure 2 shows MSEC (Eq. 6) and MSECV based on LOOCV (Eq. 7) for the 100 calibration samples. Figure 2 also shows the MSEP (Eq. 8) for the 432 prediction samples. Figure 2a shows the *MSE*s for the first 50 components, whereas Fig. 2b is a zoom-in of Components 4 to 15. As can be seen, the cross-validation indicates five components as a good choice, but prediction ability for four components is only slightly less precise. Also, the MSEP indicates that five components is a good choice in this case. The MSEC results show that the random error (Eq. 9) is quite constant after five components. This indicates that regardless of where one decides to estimate for random error, it will be approximately the same as long as more than four components are chosen. As an estimate of the random error, $\sigma^{2}$, we will use the MSEC for 50 components. It is very unlikely that there is any additional information regarding protein in components further out, which represent extremely small variability in the spectra.

**Figure 3.**
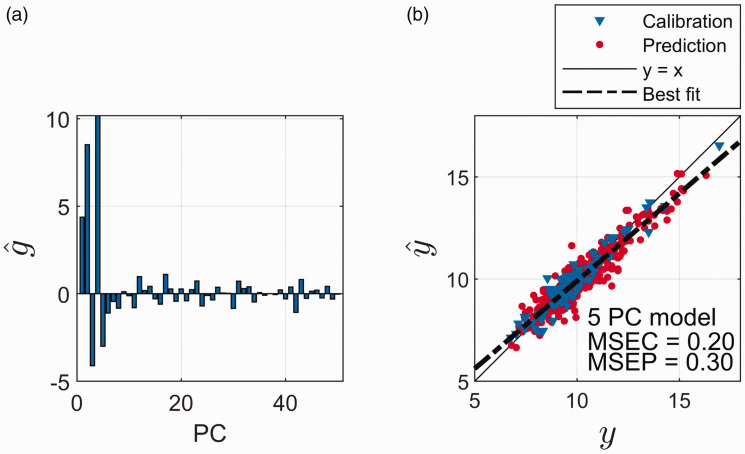
(a) Estimated regression coefficients (g^) for the different principal components (PC). The regression coefficients are estimated using the calibration samples only. (b) Measured (*y*) versus predicted (y^) values of protein for calibration samples (blue) and prediction samples (red) using a five-PC model. The mean squared error of calibration (*MSEC*) is 0.23 and the mean squared error of prediction (*MSEP*) is 0.29.

The estimated regression coefficients, g^ (Eq. 5), for the different components are presented in Fig. 3a. The first five components are strongly dominating, with a few significant ones further out. Significance is here defined according to a standard *t*-test, testing whether the slope-term (univariate regression) between **y** and the individual columns of **U** (Eq. 4) is different from zero, with significance level 0.01. The predicted protein values, y^, are plotted against the measured protein values, **y**, for a five-component PCR model, in Fig. 3b. In Fig. 3b, the least square bias effect described above is seen as a systematic tendency on top of the random noise. Larger values of **y** are in general underestimated and smaller values are in general overestimated. This is clear from the orientation of the y=x line relative to the best fit (Fig. 3b). In the supplemental material (Figure S3), the least squares bias effect is calculated (based on Eq. 12) for the 100 calibration samples. As can be seen, this follows nicely the general bias trend in Fig. 3b.

**Figure 4.**
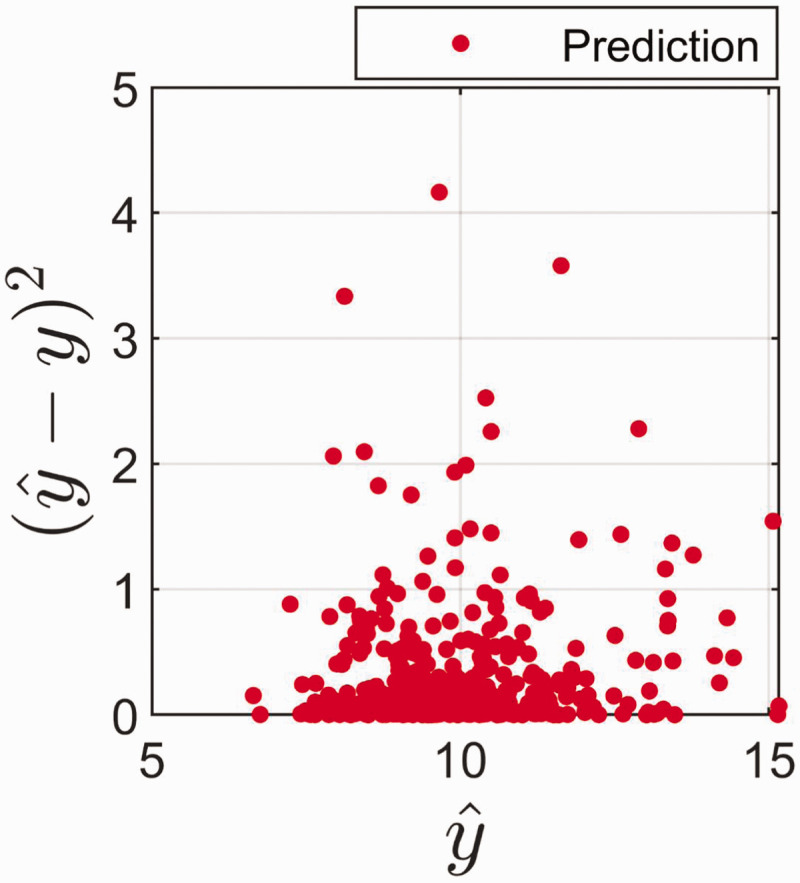
True error (y^-y) 2 as function of the estimate (y^) for prediction samples.

The phenomenon that the same predicted value can have vastly different prediction error is illustrated in Fig. 4. Figure 4 shows that the true error fluctuates between exceedingly small and exceptionally large errors for all values of the predictions. The same tendency is seen if we use Eq. 9 for prediction error instead of the true errors (data not shown). This may look a bit surprising, but it is an effect of the fact that a predicted value of *y* can be a function of quite different configurations of **x** within the model space.

**Figure 5.**
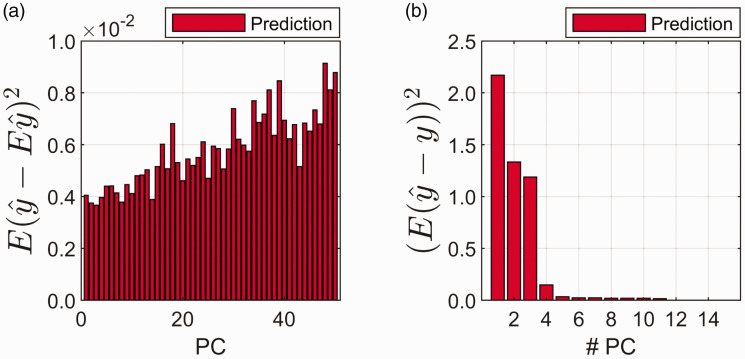
(a) Average variance contribution (E(y^-Ey^) 2) of prediction samples for each principal component (PC). (b) Average squared bias contribution ((E(y^-y)) 2) for the prediction samples as the number of principal components (# PC) increases in the model.

The variance contribution (Eq. 10) and the square of the bias contribution (Eq. 11) are presented in Figs. 5a and 5b, respectively. In Fig. 5a, the average variance contribution is shown per principal component (prediction samples only, i.e., data did not take part in fitting the PCR model). In Fig. 5b, the average (again over prediction samples only) squared bias is shown per model complexity (i.e., the bias contribution at five principal components corresponds to the bias for a five-component model). The true bias is unknown since **g** is unknown, but the bias is here estimated using the significant g^ values from 1 up to 15 components. This limit of 15 components is chosen in order to avoid too much noise from components further out to take part in the formula. These components are also, in most cases, of little relevance for the constituent of interest. As can be seen, the average variance contribution (Fig. 5a) increases and the average bias (Fig. 5b) decreases as the number of components increases. This corresponds exactly to the general principle in Fig. 1, bias decreases and random error increases with increasing model complexity. It is observed that the variance increases slowly with increasing number of components. The bias drastically decreases until the optimal number of components is reached and then it stays relatively low and constant. This behavior of variance and bias with increasing number of components is usually observed in practice.^16^

**Figure 6.**
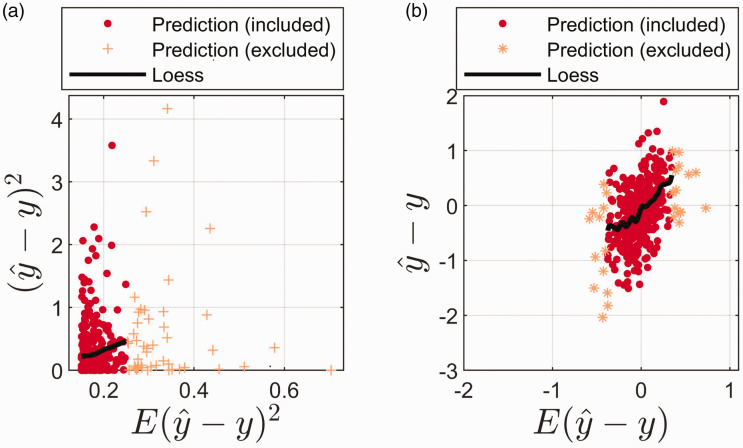
(a) Estimated prediction error versus true error for each specific sample in the prediction dataset. The expected prediction error (E(y^-y) 2) is given by Eq. 9 and the true error is given by (y^-y) 2. (b) Estimated bias versus true bias for each specific sample in the prediction dataset. The estimated bias (E(y^-y)) is given by Eq. 11 and the true bias is given by (y^-y). Loess is used for estimating the average tendency. Only samples marked with a red circle, Prediction (included), were included when estimating Loess. Samples marked as Prediction (excluded) were excluded when estimating the average tendency using Loess.

Figure 6a shows the true squared prediction errors plotted against the estimated sample-specific prediction errors (Eq. 9). Figure 6b shows the sample-specific true bias (y^-y) plotted against the estimated bias (Eq. 11). Both Figs. 6a and 6b represent the prediction dataset (i.e., the data did not take part in fitting the PCR model). Loess regression (0.5 bandwidth; second-order polynomial model) is used for estimating the average tendency in the two plots. Only samples marked as “Prediction (included)” were included during Loess estimation, in Figs. 6a and 6b. Samples marked as “Prediction (excluded)” did not take part in the Loess estimation. Figure 6a shows that the estimated average prediction ability follows quite closely the value from the formula, but as also seen, the distribution around the average varies quite a lot with the size of the error. Many values are quite small, but some are also large both for small and large values of the formula. The similarity between the average measured error and the formula supports the choices made above regarding estimate of bias (g^) and the random error ($\sigma^{2}$). Figure 6b shows that there is a clear relation between the average bias estimate and the average true bias observed. The Loess estimate shows approximate linearity, between estimated and true bias, with a slope of ∼1. This clearly indicates that the bias formula calculated as described makes sense for estimating the true bias. In Fig. 6b (like in Fig. 6a), the distribution around the average varies quite a lot, indicating that the uncertainty of the bias estimate can be large. Nevertheless, the size of the values also emphasizes the need for not always neglecting the bias in the formula for prediction error, although in this case the bias is smaller and less important than the variance contribution. The average difference between the true and estimated bias is −0.01. Hence, in this case, the theoretical formula slightly overestimates the bias. The variance of the true bias is 0.29 and the variance of the estimated bias is 0.04. This difference is due to random fluctuations in the measured bias. We did not find any relation between the estimated bias (nor the true bias) and predicted values (y^). This suggests that the bias estimate is not a function of the position in the prediction space (data not shown).

The results for a four-component PCR model are presented in Figure S4. The results show larger prediction errors for the four-component model (Figure S4a) as compared to the prediction errors for the five-component model (Fig. 3b). This is due to a larger bias in the four-component model (Figure S4b) as compared to the five-component model (Fig. 6b). The larger bias for the four-component model is a result of the large g^ value for Component 5 (Fig. 3a), which will take part in the bias contribution in the four-component model. Also, for the four-component model, the prediction ability follows the estimated prediction ability given by Eq. 9. The results for the four-component model are similar to the results for the five-component model (Fig. 6a) with a clear indication of larger variance around larger squared errors (results not shown). The difference is that in the case of a four-component model, the bias contribution is stronger.

## Conclusion

The present paper has demonstrated how the bias and variance contribute to the true prediction errors. In addition, a distinction between different bias definitions has been given and it has been demonstrated how they contribute to the overall bias of a predictor. The results indicate that the formulae for prediction error for the PCR method can be used to assess average sample dependent prediction ability, but they also show that the variability around the average values can be substantial. This means that the uncertainty of the estimate can be quite large. In addition, it has been demonstrated that the bias contribution from the PCR formulae should not always be neglected completely. We recommend estimating the regression coefficients in the PCR model and the contribution from the bias from the prediction error formulae and incorporate the bias in the overall estimation error if needed.

## Supplemental Material

sj-pdf-1-asp-10.1177_0003702820913562 - Supplemental material for Sample-Specific Prediction Error Measures in Spectroscopy Click here for additional data file. Supplemental material, sj-pdf-1-asp-10.1177_0003702820913562 for Sample-Specific Prediction Error Measures in Spectroscopy by Carl Emil Eskildsen and Tormod Næs in Applied Spectroscopy

## Supplemental Material

The supplemental material mentioned in the text, consisting of Figures S1–S4, is available in the online version of the journal.
