# Supplementary material for: Sample-Specific Prediction Error Measures in Spectroscopy
Source: Appl Spectrosc. 2020 May 26;74(7):791–8. doi: 10.1177/0003702820913562 (PMC7745614; doi:10.1177/0003702820913562)
Supplement: sj-pdf-1-asp-10.1177_0003702820913562 - Supplemental material for Sample-Specific Prediction Error Measures in Spectroscopy [file sj-pdf-1-asp-10.1177_0003702820913562.pdf]

Sample-Specific Prediction Error Measures in Spectroscopy

Carl Emil Eskildsen[1,2]* and Tormod Næs[1]

[1]Nofima AS, Norwegian Institute for Food, Fisheries and Aquaculture Research, Osloveien 1, NO-1430, Ås, Norway

[2]Institute for Biodiversity and Ecosystem Dynamics, University of Amsterdam, Science Park 904, NL-1098 XH Amsterdam, The Netherlands

*Corresponding author email: c.e.eskildsen@uva.nl

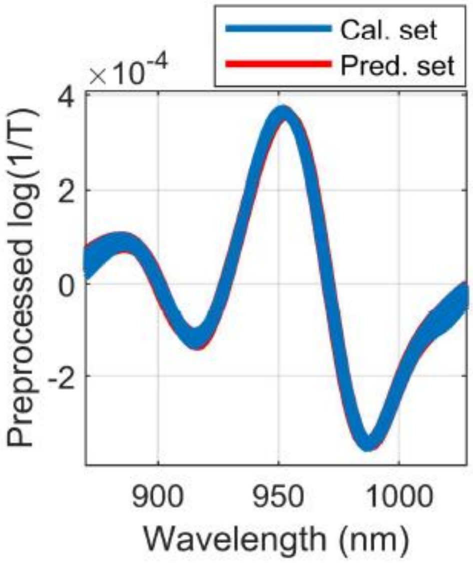

Figure S1. Near-infrared spectra obtained on individual wheat kernels. Calibration (blue) and prediction (red) samples.

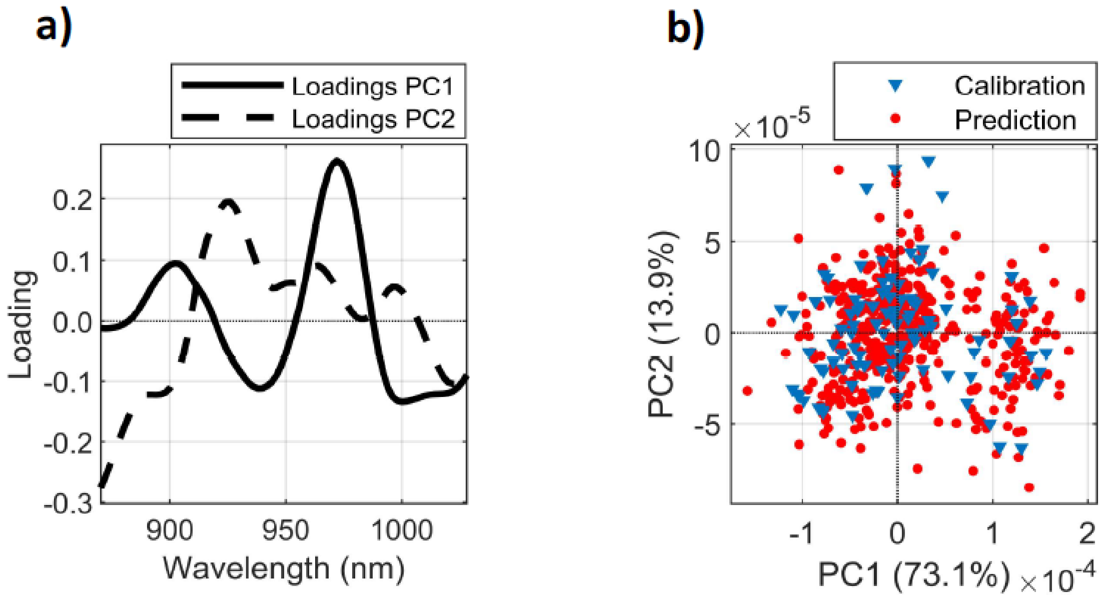

Figure S2. Singular Value Decomposition of near-infrared spectra obtained on individual wheat kernels. (a) Loadings and (b) scores for the two fist principal components (PC). Test set in red, calibration set in blue.

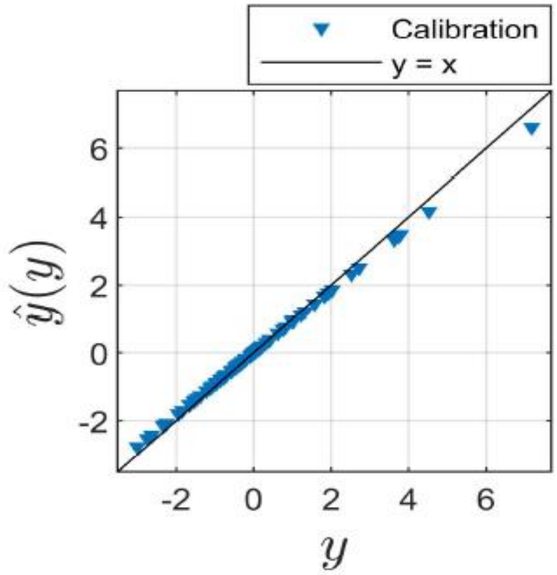

Figure S3. Illustration of the least squares (LS) bias as a function of y.

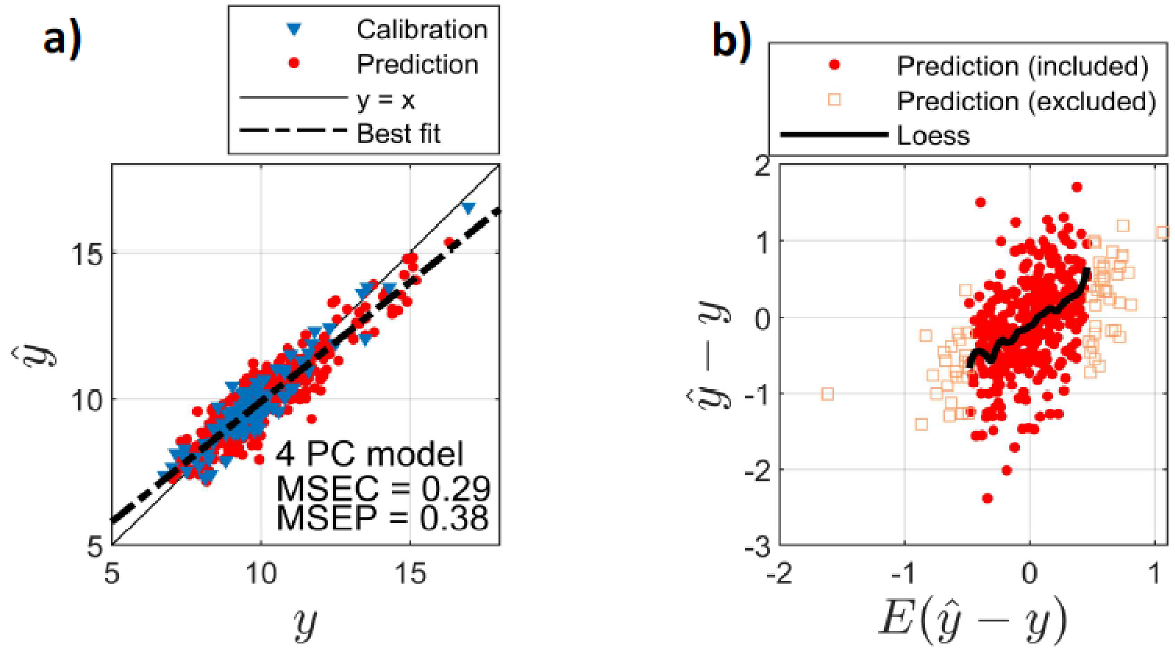

Figure S4. Underfitted PCR model (four PCs). (a) Measured (y) versus predicted ($\hat{y}$) values of protein for calibration samples (blue) and prediction samples (red). Mean squared error of calibration (MSEC) is 0.33 and mean squared error od prediction (MSEP) is 0.33. (b) Estimated bias versus true bias for each specific sample in the prediction dataset. The estimated bias ($E(\hat{y} - y)$) is given by Eq. 11 and the true bias is given by ($\hat{y} - y$). Loess is used for estimating the average tendency. Only samples marked with a red circle, Prediction (included), were included when estimating Loess.
